# Sleep quality among non-hospitalized COVID-19 survivors: a national cross-sectional study

**DOI:** 10.3389/fpubh.2023.1281012

**Published:** 2024-02-05

**Authors:** Huong Thi Xuan Hoang, Wing Fai Yeung, Quyen Thi Mai Truong, Cuc Thi Le, Anh Thi My Bui, Quang Vinh Bui, Quyen Thi Le Le, Linh Ha Quach

**Affiliations:** ^1^Faculty of Nursing, Phenikaa University, Hanoi, Vietnam; ^2^School of Nursing, The Hong Kong Polytechnic University, Hong Kong SAR, China; ^3^Hoan My Da Lat Hospital, Dalat, Vietnam; ^4^Department of Nursing and Midwifery, Hanoi Medical University, Hanoi, Vietnam; ^5^School of Preventive Medicine and Public Health, Hanoi Medical University, Hanoi, Vietnam; ^6^Hanoi Oncology Hospital, Hanoi, Vietnam; ^7^Center for Ageing Research & Education, Duke-NUS Medical School, Singapore, Singapore

**Keywords:** post COVID-19, insomnia, depression, anxiety, ISI, DASS-14

## Abstract

**Objectives:**

Insomnia is a common symptom after COVID-19 infection; however, its current evidence was among hospitalized COVID-19 patients. This study aimed to assess the prevalence of insomnia and identify its association with depression and anxiety among non-hospitalized COVID-19 recovered population.

**Methods:**

We conducted a cross-sectional online survey of 1,056 COVID-19 survivors within 6 months of initial COVID-19 infection and retrieved did not require hospitalization. The Insomnia Severity Index, and Depression Anxiety and Stress Scale-14 were used. Multivariate logistic regression was used to examine the associations between depressive and anxiety score, and participants’ insomnia level.

**Results:**

The prevalence of insomnia was 76.1%, and among those, 22.8% of participants scored for severe insomnia. One third of participants reported worse sleep quality, shorter sleep duration, and harder to fall asleep, half reported more awaken nights after COVID-19 infection. Participants with depressive (OR 3.45; 95%CI 1.87–6.34) or anxiety (OR 3.93; 95%CI 2.52–6.13) had significantly higher odds of developing insomnia. Other risk factors of insomnia included pre-existing chronic conditions and higher education level, while COVID-19 symptoms and duration were not significantly associated.

**Conclusion:**

Our study highlights the substantial burden of insomnia among non-hospitalized COVID-19 survivors and the significant association of depression and anxiety on the development of this long-term effect of COVID-19. These findings underscore the need for comprehensive interventions that address both sychological and sleeping health in this population.

## Introduction

Since late 2019, COVID-19 pandemic had reached every region of the world and infected over 767 million people globally (1). As of mid-2023, about 90% of COVID-19 infected cases had recovered from the disease (2). Most common symptoms reported during the acute phases of COVID-19 were cough, fatigue, fever, dyspnea, musculoskeletal disorders, gastrointestinal symptoms, anosmia, dysgeusia, and vertigo (3–5). As the results, COVID-19 patients face many unpleasant symptoms after recovering. Post COVID-19 condition, commonly known as long COVID has been defined as symptoms experienced by COVID-19 patients after the initial SARS-CoV-2 infection, these symptoms can affect anyone exposed to SAR-CoV-2, regardless the severity of original symptoms (6).

Although the pandemic is expected to become an endemic disease, prolonged physical and psychological problems after COVID-19 infection should be considered an important public health issue that needs addressing promptly. Among 200 different symptoms after COVID-19 infection, insomnia is one of the most common symptoms (7–9). Two recent systematic reviews have revealed a wide prevalence of insomnia among COVID-19 recovered patients ranging from 5.4 to 66.67% (8, 9). In general population, when being measured by the standard scale, this prevalence ranged from 10.1 to 66.67% (10–12), while using the standardized classification system of diagnosis codes (ICD-10) it ranged from 5.4 to 36.5% (13, 14). Longitudinal studies among COVID-19 patients after 6 months discharged from hospital reported the proportion of insomnia ranging widely from 10.1 to 45.1% (12, 15, 16).

Several factors have been reported as high-risk factors for insomnia among COVID-19 survivors, such as being female, younger age, higher employment and education status (17). Fu et al. and Campo-Arias et al. both showed anxiety, depression, post-traumatic stress disorders were associated with higher incidence of post-COVID-19 insomnia in general population (15, 18). Research has demonstrated that poor mental health is closely tied to inadequate sleep conditions. Additionally, patients infected with COVID-19 are at risk of developing anxiety and depression, as a result of the severity and isolation. In addition, chronic disease such Obstructive Sleep Apnea (OSA) impact the development of incident glycemic, neurocognitive impairment, and abnormal functional pulmonary changes that persist up to 1 year after COVID-19 recovering (19). Considering the extended recovery period for COVID-19, its impact on patients’ mental health may lead to further negative effects on their sleep quality.

While research has been conducted on sleep issues among those who have recovered from COVID-19, there is still limited information on sleep disruptions in COVID-19 survivors, regardless of whether they were hospitalized or not (17). Currently literature focused on COVID-19 hospitalized patients, which the environment of their treatment and quarantine would differ greatly from those with milder symptoms of COVID-19. Hence, this would impact the COVID-19 survivors who quarantined and received treatment out of hospital, both in terms of psychological impacts and long COVID-19 symptoms. Therefore, we conducted this study to (i) assess the prevalence of insomnia among COVID-19 survivors with no or mild symptoms that not required hospitalization in the recovery period (6 months), and (ii) identify its associated factors. Data from this study would highlight the extent of the problem and inform the development of future intervention trials.

## Methods

### Study design

A cross-sectional survey was conducted.

### Participants

Participants were COVID-19 survivors (recovered as confirmed by Polemerase Chain Reaction test) in Vietnam general population. Participants were 18 years old or above who had recovered from COVID-19 within 6 months, and not require hospitalization due to COVID-19 symptoms. Any participants with medical history of diagnosed insomnia and/or psychiatric disorder prior to infection (self-report) were excluded from the study.

### Measurements

#### Data related to COVID-19 severity

Collected variables on socio-demographic characteristics were age, sex, marital status, employment status, education level, occupation, and pre-existing chronic conditions (Yes if participants answered “Yes” for cardiovascular diseases, cancer, metabolic diseases (i.e., diabetes, high blood lipids), immunocompromised and/or allergic conditions, other). Participants were asked to rate the severity of their COVID-19 infection (asymptomatic or symptomatic), and number of days infected (calculated by days from the first positive COVID-19 test to the first negative COVID-19 test).

#### Insomnia

The Vietnamese version of the Insomnia Severity Index (ISI) was used to measure insomnia severity among participants. The ISI contains seven items reporting the nature, severity, and impact of insomnia. Each question is given a 5-point Likert scale from 0 (no problem) to 4 (very severe problem), which yields a final score ranging from 0 to 28 for each participant, higher score indicating more severe insomnia level. A score of 10 and above indicated the participants had insomnia. This cut-off point allows the most optimal sensitivity (86.2%) and specificity (87.7%) to detect insomnia in the community (20). Insomnia severity is interpreted as follows: mild insomnia (11–14), moderate insomnia (ISI score of 15–21) and severe insomnia (ISI score of 22–28) (20). In the current study, the ISI had high reliability with Cronbach’s Alpha = 0.93.

In addition, the respondents were also asked to compare their sleep quality, sleep initiation, and total sleep duration in the recent 2 weeks with the time before confirming COVID-19 infection on a 3-point Likert-like scale (3 “worse/less than before,” 2 “about the same,” 1 “better/more than before”).

#### Other symptoms

The Vietnamese version of Depression, Anxiety, and Stress Scale (DASS-14) was used to measure participants’ mental health. DASS-14 is the modified version of the DASS-21. It is a self-report tool containing 14 items that measure the level of depression and anxiety in the preceding week of participants (7 items per subscale). Participants answered the items using a 4-point Likert scale ranging from 0 (Did not apply at all) to 3 (Applied very much, or most of the time). The total score was calculated by the sum of all items multiplied by two, ranging from 0 to 42 for each of the sub-scale. A cut-off point of 33 on each sub-scale was used to create two binary variables of “Participants with depressive/anxiety symptoms” or without depressive/anxiety symptoms (sensitivity 79.1% and specificity 77.0%) (21). In this study, the internal consitency was reported to be high, with the overall scale having Cronbach’s Alpha = 0.9, the depression subscale’s Cronbach’s Alpha = 0.9, and the anxiety subscale’s Cronbach’s Alpha = 0.81.

### Sample size calculation

Sample size calculation was based on the estimated prevalence of insomnia (50%) with a 3% margin of error (10, 14). An estimated sample of 1,068 was suggested under the 95% confidence interval.

### Data collection

The questionnaire was created using Qualtrics CoreXM. The survey was opened from June to September 2022. An open invitation to participate was sent to all the member of COVID-19 patients’ network (organized by the Vietnamese government) to approach infected participants. The invitation was sent to either email address or social account (Facebook and Zalo – two most popular social media platforms) of the potential participants.

### Data analysis

Data were extracted from the Qualtrics into Stata 15.0 for cleaning and analysis. First, we tabulated participants’ sociodemographic, health, sleep quality assessment, depressive and anxiety level, stratified by insomnia score and binary level. Then the Wilcoxon rank-sum, Pearson’s chi-squared tests and Fisher’s exact tests were used to detect statistically significant differences between participants’ characteristics. Thirdly, we included depressive level, anxiety level, and each covariate into the univariate logistic regression to estimate the association with participants’ insomnia status. The univariate and multivariable logistic regression was employed to estimate odds ratios (ORs) and 95%CIs of the associations between depressive and anxiety level, and participants’ insomnia, controlling for all covariates adjusted for participants’ socio-demographic characteristics, the severity of their COVID-19 infection, and number of days infected.

## Results

The research team sent out 34,850 invitation to targeted population and a total of 1,090 participants responded to the survey. However, 34 participants did not complete the ISI and DASS-14 leaving the final sample size of the study was 1,056. Table 1 shows descriptive values of sociodemographic characteristics, COVID-19 infection, and mental health indicators of participants, summarized by ISI score and stratified by insomnia status. Majority of participants were female (68.5%), mean age of 33.6 ± 10.1, married (64%), and had received university education (68.7%). 20.1% of our participants were healthcare workers, 16.1% were students, and 3.4% were not working at time of survey. In addition, 11.7% of them had pre-existing chronic conditions (cardiovascular diseases: 4.5%, metabolic disorders: 3.5%, cancers: 2.2%, and other chronic diseases such as degenerative spine, adiposis hepatica, gout: 1.5%). Participants who were female, divorced/widowed, had post-graduate education, not currently working, or had chronic diseases scored higher ISI score on average than their counterparts, but only those with chronic conditions had significantly higher score (18.46 ± 6.03, *p* < 0.01) than those without.

**Table 1:** Descriptive analyses of sociodemographic characteristics, COVID-19 infection, and mental health indicators of participants.

	Total (*n*, %)	ISI score		Value of *p*^a^	Insomnia participants (n, %)	Value of *p*^b^
		Mean	SD			
*N*	1,056	15.1	6.0		804 (76.1)	
Sociodemographic characteristics						
Gender				0.95		0.48
Male	329 (31.2)	14.97	6.1		253 (31.5)	
Female	723 (68.5)	15.11	6.1		547 (68.0)	
LGBT	4 (0.4)	14.75	4.11		4 (0.5)	
Age (Mean, SD)	33.6 (10.1)	--	--		33.5 (10.4)	
Marital status				0.35		0.04*
Single	358 (33.9)	15.4	5.8		289 (35.9)	
Married	676 (64.0)	14.8	6.1		498 (61.9)	
Divorced/ Widowed	22 (2.1)	15.4	6.1		17 (2.2)	
Education level				0.06		0.07
Formal education	125 (11.8)	14.2	6.13		85 (10.6)	
University	725 (68.7)	15.0	5.9		562 (69.9)	
Post-graduate	206 (19.5)	15.8	6.2		157 (19.5)	
Occupation				0.08		0.10
Healthcare worker	212 (20.1)	14.0	5.8		147 (18.3)	
Other occupation	638 (60.4)	15.3	6.0		493 (61.3)	
Student	170 (16.1)	15.2	5.9		136 (16.9)	
Not currently working	36 (3.4)	15.8	6.4		28 (3.5)	
Pre-existing chronic condition				0.007		<0.001*
No	932 (88.3)	14.62	5.9		691 (85.9)	
Yes	124 (11.7)	18.46	6.03		113 (14.1)	
COVID-19 infection						
Having symptoms of COVID-19				0.71		0.6
No	88 (8.3)	13.9	5.45		69 (8.6)	
Yes	968 (91.7)	15.2	6.1		735 (91.4)	
Number of SARS-CoV-2 infected weeks (Mean, SD)	11.2 (4.3)	--	--		--	
Mental health indicators						
Depression score (Mean, SD)	6.4 (8.3)	--	--		7.8 (8.8)	
Depression level				<0.001*		<0.001*
No depressive symptoms	765 (72.4)	13.5	5.4		528 (65.7)	
With depressive symptoms	291 (27.6)	19.1	5.7		276 (34.3)	
Anxiety score (Mean, SD)	7.6 (8.0)	--	--		9.2 (8.3)	
Anxiety level				<0.001*		
No anxiety symptoms	617 (58.4)	12.4	4.6		397 (49.4)	
With anxiety symptoms	439 (41.6)	18.7	5.9		407 (50.6)	

^a^Value of *p* was calculated to differentiate between ISI score of different sociodemographic characteristics, COVID-19 infection, and mental health indicators.

^b^Value of p was calculated to differentiate between insomnia status (Yes/No) of different sociodemographic characteristics, COVID-19 infection, and mental health indicators.

Regarding COVID-19 infection, 92% of participants were symptomatic during their period of infection (on average 11.2 ± 4.3 weeks). These symptomatic participants also scored higher on ISI (15.2 ± 6.1), yet no statistically significant was found compared to asymptomatic participants. The mean score of depression was 6.4 ± 8.3, and the mean score of anxiety was (7.6 ± 8.0). This resulted in 291 (27.6%) and 439 (41.6%) of participants with relevant depressive and anxiety symptoms, respectively. Participants with depressive symptoms (19.1 ± 5.7) and with anxiety symptoms (18.7 ± 5.9) also had significantly higher ISI score than those without (13.5 ± 5.4 and 12.4 ± 4.6, respectively, *p* < 0.001).

**Figure 1:**
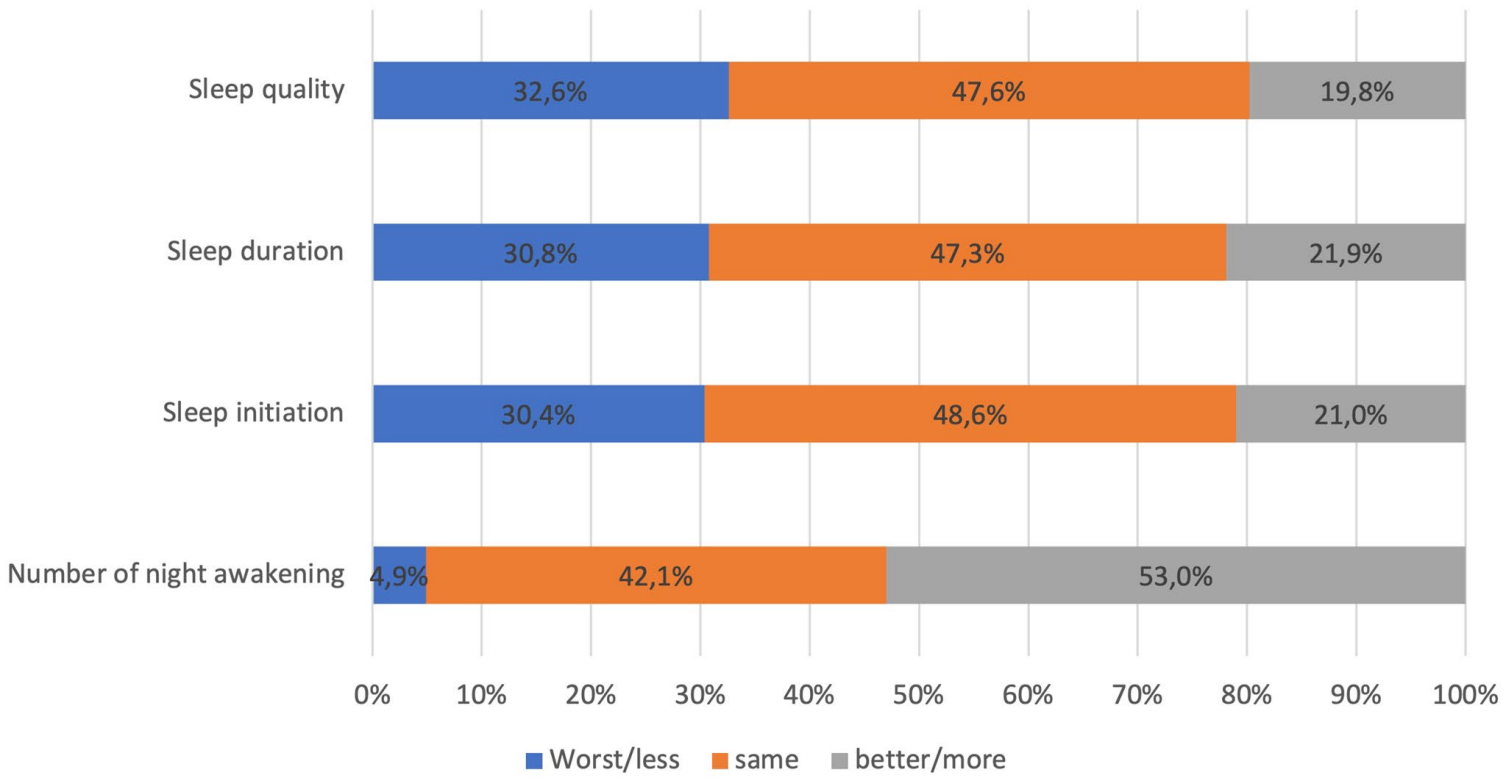
subjective assessment of some sleep parameters after COVID-19 infection of 1,056 participants.

The average ISI score of the participants was 15.1 ± 6.0. The prevalence of insomnia was 76.1% (804/1056 participants) (mean score of ISI among insonnia participants = 17.32 ± 5.1). Among those who experienced insomnia, 41.5% reported moderate, and 22.8% reported severe insomnia. Characteristics of insomnia participants were comparable to the whole sample size. In addition, there were significant differences in marital status (*p* < 0.04), pre-existing chronic condition (*p* < 0.001), depression and anxiety level (*p* < 0.001) between participants with and without insomnia. The participants with insomnia had higher depression scores (7.8 ± 8.8) and anxiety scores (9.2 ± 8.3) than the average scores of the entire sample. Figure 1 shows participants’ subjective assessment of sleep condition after COVID-19 infection. About one third of participants consistently reported their sleep quality, duration, and initiation was worse than before they were infected with COVID-19. More than half of participants (52.9%) reported higher number of night being awake after recovering from COVID-19 compared to before being infected.

Table 2 shows univariate and multivariable logistic regression of factors associated with insomnia among participants. In univariate regression, participants who were married [Odds ratio (OR) 0.68,95% CI: 0.49–0.91, *p* < 0.05] and had university education (OR 1.62, 95% CI: 1.07–2.45, *p* < 0.05) had significantly lower odds of having insomnia than those who were single or had formal education. Participants who were students (OR 1.77, 95% CI: 1.10–2.85, *p* < 0.05) had significantly higher odds of having insomnia than healthcare workers. Participants who had pre-existing chronic conditions (OR 3.58, 95% CI: 1.90–6.77, *p* < 0.001) had significantly higher odds of insomnia after their COVID-19 infection than those without chronic diseases. Concerning COVID-19 infection, there was no significant likelihood of having insomnia post-COVID-19 infection between symptomatic status or time of infection. On the other hand, participants with depressive symptoms (OR 8.26, 95% CI: 4.80–14.19, *p* < 0.001) or anxiety symptoms (OR 7.05, 95% CI: 4.74–10.47, *p* < 0.001) had significantly higher proportion of insomnia than those without.

**Table 2:** Univariate and multivariable logistic regression of factors associated with insomnia among 1,056 participants.

	Univariate analysis		Multivariable analysis	
	OR (95% CI)	Value of *p*	OR (95% CI)	Value of *p*
Sociodemographic characteristics				
Gender				
Male	Ref		Ref	
Female	0.93 (0.69–1.27)	0.662	0.86 (0.61–1.21)	0.376
LGBT	--		--	
Age	0.99 (0.98–1.01)	0.526	1.01 (0.99–1.03)	0.359
Marital status				
Single	Ref		Ref	
Married	0.68 (0.49–0.91)	0.012*	0.66 (0.41–1.06)	0.085
Divorced/ Widowed	0.81 (0.29–2.28)	0.692	0.75 (0.24–2.40)	0.633
Education level				
Formal education	Ref		Ref	
University	1.62 (1.07–2.45)	0.022*	1.85 (1.10–3.10)	0.020*
Post-graduate	1.51 (0.92–2.47)	0.103	1.60 (0.91–2.82)	0.102
Occupation				
Healthcare worker	Ref		Ref	
Other occupation	1.50 (1.06–2.12)	0.201	1.62 (1.10–2.41)	0.015
Student	1.77 (1.10–2.85)	0.019	2.54 (0.96–6.74)	0.062
Not currently working	1.55 (0.67–3.58)	0.307	1.16 (0.62–2.14)	0.640
Pre-existing chronic condition				
No	Ref		Ref	
Yes	3.58 (1.90–6.77)	<0.001*	2.96 (1.50–5.84)	0.002*
COVID-19 infection				
Having symptoms of COVID-19				
No	Ref		Ref	
Yes	0.87 (0.51–1.47)	0.602	0.64 (0.36–1.13)	0.123
Number of SARS-CoV-2 infected weeks	0.99 (0.96–1.03)	0.753	0.99 (0.96–1.04)	0.984
Mental health indicators				
Depression level				
No depressive symptoms	Ref		Ref	
With depressive symptoms	8.26 (4.80–14.19)	<0.001*	3.45 (1.87–6.34)	<0.001*
Anxiety level				
No anxiety symptoms	Ref		Ref	
With anxiety symptoms	7.05 (4.74–10.47)	<0.001*	3.93 (2.52–6.13)	<0.001*

Models were estimated by univariate and multivariable logistic regression to examine the association with the prevalence of insomnia. Univariate regression included each variable in the model. Multivariable regression included all variables in the model.

The multivariable regression confirmed the significant association between education level, pre-existing chronic conditions, and the prevalence of insomnia. Participants who had university education had 1.85-fold (95% CI: 1.10–3.10) increase in the odds of experiencing insomnia after their COVID-19 infection compared to those with formal education (primary, junior high, and high school). Participants with chronic conditions had 2.96 (95% CI: 1.50–5.84) times higher odd of having insomnia than those without. After adjusting for covariates, healthcare workers had significantly higher odd of having insomnia (OR 1.62, 95% CI: 1.10–2.41) than workers in other professions, but no significances as compared to those who were not working or were students. Multivariable regression also showed significantly higher odds of having insomnia in participants with depressive symptoms (OR 3.45, 95% CI: 1.87–6.34) and anxiety symptoms (OR 3.93, 95% CI: 2.52–6.13) than those without, but these associations were weaker than in the univariate model. The correlations between insomnia, anxiety, and depression were strong to moderate and significant (*r* rank from 0.5 to 0.8, *p* = 0.01) (Table 3).

**Table 3:** Correlation between insomnia, anxiety, and depression score.

	ISI score	Anxiety- D	Depression - D
ISI score	1		
Anxiety – D	0.541**	1	
Depression - D	0.796**	0.482**	1

^**^Correlation is significant at the 0.01 level (2-tailed); ISI, insomnia severity index; Anxiety – D, anxiety score measured by DASS-14; Depression – D, depression score measured by DASS-14.

## Discussion

Results from the study indicated that more than 75% of participants had insomnia, which is remarkably higher than previously reported among general population (10–20%) (22, 23). This proportion is also much higher than the prevalence of insomnia among hospitalized COVID- 19 survivors presented in previous systematic reviews (12–47%) (4, 24, 25). This can be explained by the selection criteria (participants had recovered from COVID-19 within 6 months) so insomnia was expected to be more prevalent. Newly covered from an infection, especially a novel viral epidemic, can cause survivors to have increased risk of chronic pain, chronic health, and psychiatric disorders, which can lead to increased insomnia (26). As in our findings, we also found that elevated depressive and stress symptoms associated with insomnia risk. In addition, insomnia is often underreported in general population (27) and often higher in people with chronic diseases or health problems, hence, the findings can reflect a heightened sense of personal health among COVID-19 survivors regarding their sleep quality. As insomnia is also one of the most frequently reported symptoms among COVID-19 survivors (25), public health researchers should expect higher prevalence of insomnia and sleep problems reported among this population, which can remain among one-third recovered patients till 1 year after infection according to a longitudinal research (16).

It was observed that more than two-third of participants reported insomnia, however, only one-third reported their sleep quality, duration, and ability to initiate sleep were worse than before they were infected with COVID-19. Notably, more than half of participants reported an increase of night awakenings. Insomnia was related to the amount of delta sleep (the deepest form of sleep) and improper estimation of sleep parameters such as the total sleep time, the sleep latency, and wakening after sleep onset (28, 29). As such, an intervention that help participants to change their perception of sleep parameters may be helpful to improve insomnia.

In general population, being single or divorced have been found to be positively associated with insomnia (30–33). Other studies have also shown that the proportion of COVID-19 survivors with insomnia is higher among single or divorced individuals than among those with others marital status, which corresponds with the results of the present studies (9, 18, 34). We did not find a significant risk of insomnia of healthcare workers compared to other occupation, which is contradicting previous studies (35). As there are various aspects of healthcare workers – direct involvement with patients, shift working, clinical work versus public health work – that can impact their insomnia level. At time of the survey, COVID-19 epidemic in Vietnam was more under control, so healthcare workers might not be subjected to additional stress of the pandemic. Also in 2022, many companies, schools, and workplaces reopens, and the readjustment from working from home to office working might put a higher toll on sleep health of participants of other occupations than healthcare workers – who work in hospital throughout the pandemic.

Chronic diseases had increased the severe COVID-19 infection (36, 37). Additionally, patients with chronic illnesses are more likely to develop insomnia (38). Even though there has no conclusive on the reciprocal relationship between pre-existing chronic diseases and insomnia after COVID-19, it is possible that the severity of the infection and/or the mental burden of the disease could contribute to the onset of insomnia. The current study excluded people with a history of insomnia prior to expose with COVID-19. As such the results confirm the vulnerability of individuals with chronic diseases to insomnia after infection. This highlights the need for health monitoring and services specifically designed for high-risk groups following their infection.

This study confirmed anxiety and depression were significant associated with insomnia among recovered COVID19 patients, which is in line with previous studies (10, 36, 37, 39, 40). As COVID-19 infected patients were at higher risk of developing mental health problems (41), the psychological burden of being infected and quarantine would much likely to negatively impact their sleep quality even after their infection. Insomnia and sleep disturbances were also often cited as one of the co-occurrence symptoms of depression and anxiety. The symptom cluster of insomnia, depression, and anxiety has been reported in different populations (42–46). Therefore, more researchs must be implemented to further investigate the relationship between this three symtopms during the post-COVID-19 period.

In this study, the correlations between the insomnia, depression, and anxiety were strong to moderate in the correlational analysis. In addtion, bidirectional relationships between these three symptoms had been emphasized in systematic reviews indicating that an intervention to improve sleep should also address depression and anxiety (47, 48). This supports the use of antidepressant and/or benzodiazepine as potential treatments for improving sleep in this population when anxiety and/or depression are present, striking a good balance between the potencial possitive benefit and adverse effects and polypharmacy. However, given the potential adverse effects of pharmacological treatment non-pharmacological treatments, such as self-acupressure, cognitive behavioral therapy, mind subtraction meditation, and Cranial Electrotherapy Stimulation should be considered as they may alleviate the three symptoms (49–53). Public health agencies should consider these interventions to mitigate the long-term negative impact of the pandemic on its growing infected population.

The large sample size and the used of standardized questionnaires to measure study’s outcomes are strengths of the study. Authors acknowledge that the current study has several limitations. Since this is a cross-sectional study, the direct causation or the impact of anxiety and depression on insomnia cannot be made. In addition, due to the online collecting data and convenient sampling method, recalled bias and selection bias may occur. However, due to the situation in Vietnam at that time, collecting data via electronic invitation and convenience sampling was the most efficient and feasible strategy. In addition, some sleep parameters, vaccination status, and physical symptoms of COVID-19 were not measured in this survey but subjected to reporting bias, which can confound the findings. As severity levels and COVID-19 vaccination status might impact COVID-19 patients’ health and well-being, inclusion of other COVID-19 infection conditions might improve the accuracy of our findings and detect the high-risk population for insomnia among COVID-19 survivors. At time of survey, COVID-19 vaccination rate in Vietnam for fully vaccinated dose was at least 70% at all provinces and cities (54) so we suspect this lack of data would impact on our findings.

## Conclusion

The results of the current study confirm the high prevalence of insomnia COVID-19 survivors with none or mild symptoms who did not require hospitalization. Pre-existing chronic conditions, depression, and anxiety were associated with higher risk of developing insomnia during recovery of COVID-19. Our findings add to current literature on insomnia after COVID-19 infection and underscores the crucial need to implement comprehensive interventions to address the psychological and sleep health of COVID-19 patients after recovery.

## Data availability statement

The raw data supporting the conclusions of this article will be made available by the authors, without undue reservation.

## Ethics statement

The studies involving humans were approved by the study obtained ethical approval from the Human Subject Ethics Board, Nam Dinh Nursing University. The studies were conducted in accordance with the local legislation and institutional requirements. The participants provided their written informed consent to participate in this study.

## Author contributions

HH: Conceptualization, Investigation, Methodology, Supervision, Writing – original draft, Writing – review & editing. WY: Conceptualization, Methodology, Writing – review & editing. QT: Data curation, Formal analysis, Investigation, Writing – review & editing. CL: Formal analysis, Investigation, Methodology, Writing – review & editing. CL: Formal analysis, Investigation, Methodology, Writing – review & editing. AB: Data curation, Formal analysis, Investigation, Methodology, Writing – review & editing. QB: Investigation, Methodology, Project administration, Writing – review & editing. QL: Investigation, Project administration, Writing – review & editing. LQ: Conceptualization, Formal analysis, Investigation, Methodology, Writing – review & editing.
